# Describing fluctuating indoor aerosol dust measurements with application to house dust mite allergens

**DOI:** 10.1038/s41598-020-73839-x

**Published:** 2020-10-09

**Authors:** F. E. van Boven, N. W. de Jong, M. G. L. C. Loomans, G. J. Braunstahl, R. Gerth van Wijk, L. R. Arends

**Affiliations:** 1 Department of Internal Medicine, Section of Allergology & Clinical Immunology, Erasmus Medical Center, Rotterdam, The Netherlands; 2 Department of the Built Environment, Building Performance IEQ-Health, Eindhoven University of Technology, Eindhoven, The Netherlands; 3 Department of Pulmonology, Franciscus Gasthuis & Vlietland, Rotterdam, The Netherlands; 4 Department of Pulmonology, Erasmus Medical Center, Rotterdam, The Netherlands; 5 Department of Biostatistics, Erasmus Medical Center, Rotterdam, The Netherlands; 6 Department of Psychology, Education & Child Studies, Erasmus University Rotterdam, Rotterdam, The Netherlands

**Keywords:** Environmental sciences, Immunological disorders, Statistics, Entomology

## Abstract

Measuring house dust mite aeroallergen concentrations is essential in understanding mite allergen exposure. Physically, the aerolized house dust mite faeces are part of indoor particulate matter. We studied the statistical ways of summarizing measurements of fluctuating mite aeroallergen exposure inside homes through indoor particulate matter. To study emissions from beddings, we measured the time-related airborne dust concentration after shaking a duvet. Analysis was performed both by a method based on the estimated mean and by a non-linear model. Twenty-eight studies reported a sum of concentrations; only one also reported the peak. In our four experiments on shaking a duvet (245 to 275 measurements each), the peak value was two to four times higher than the mean. The mean-based and non-linear models both predicted the sum of concentrations exactly. A 1% upper prediction bound and the non-linear model predicted the peak emission rate moderately well (64 to 92%, and 63 to 93%, respectively). Mean levels of indoor particulate matter measurements differ substantially from peak concentrations. The use of the mean is only sufficient to predict the sum of concentrations. We suggest that, mite aeroallergen measurements should include information on the peak as well as the mean.

## Introduction

Assessment of exposure to house dust mite aeroallergens is essential in determining the potential contribution to an allergic reaction^1^. Measuring such aeroallergens is possible through sampling of indoor particulate matter, followed by an assay. Indoor particulate matter may contain a spectrum of pollutants, such as: house dust mite allergens; bacteria; fungal spores; organic compounds^2^. Environmentally, indoor exposure is characterized by the peak concentration (the largest amount of mite aeroallergen that a person is exposed to at any one time) and the sum of concentrations (the total amount of mite aeroallergen that a person is exposed to during a specified time)^3,4^. In daily life, patients are exposed in their homes to repeated environmental emissions, followed by decays^5^. Modern techniques show that fluctuations in indoor particulate matter take seconds to a minute^6^, that are commonly described by the mean as well as percentiles^7^. In the field of aerobiology, such diurnal fluctuating exposure is modelled with a tailored model, such as a time series or periodic function^8^.

Methods for the measurement of airborne house dust mite particles during specific portions of the day or night, e.g. during sleep, have also been proposed^9^. The species of the mites *Dermatophagoides pteronyssinus, Dermatophagoides farinae*, *Euroglyphus maynei*, as well as the specie *Blomia tropicalis*, are important domestic sources of house dust mite allergens, which are found mainly in their faecal products^10,11^. The spherical faecal particles, with diameters of 10–40 μm^5^, partially degrade with time into smaller fragments of 1–10 μm^12^, which easily remain airborne. Most large airborne mite allergens settle rapidly in five to twenty minutes after emission^13,14^. Historically, experiments on exposure to indoor mite aeroallergens expressed variation in terms of either the mean concentrations during disturbed conditions or the mean concentrations during undisturbed conditions^5,15,16^. Sampling periods started from 20 min duration. However, from a statistical point of view, a not well-discussed topic is whether the use of mean concentrations describing mite aeroallergen concentrations can be improved by presenting more information on the peaks.

Physically, the aerolized house dust mite faeces are part of indoor particulate matter, consequently following the principles of indoor particle dynamics^3^. The dynamic behaviour of these particles, like deposition and re-suspension, are strongly related to the particle size^17,18^. This indoor particle behaviour can be described by physical models^3^. Consequently, summary-statistics have to cover the physical tendencies in indoor particulate matter, which are compatible for different types of particle compositions. In the field of aerosol exposure, the use of proxies is common, for instance like used in assessing respiratory penetration^19^. This creates the possibility for using real-time particulate matter measurements as a proxy for observing fluctuations in mite aeroallergen concentrations, with larger statistical power.

The aim of this study is to examine the summary-statistics of describing variation in dust mite allergen exposure levels inside homes through varying indoor particulate matter. Accordingly, we first search and summarize the literature on statistics of indoor aeroallergens measurements inside homes, and then experimentally study particulate matter emission from bedding, in order to evaluate the mean concentration, peak concentration, and sum of concentrations and using the methods of applied statistics.

## Methods

### Summary-statistics on mite aeroallergens in the literature

**Figure 1:**
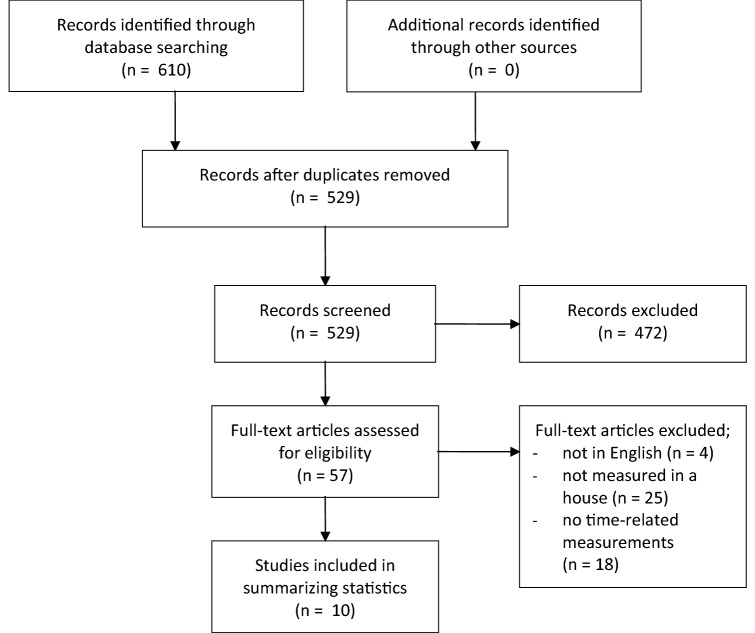
PRISMA Flow chart of the literature search.

A sample from the literature on indoor measurement of airborne mite-allergen exposure was selected from Pubmed and Web of Science by use of the keyword search-strings ‘airborne AND mite AND (allerg* OR antigen* OR exposure)’ and ‘aeroallergen AND mite AND sampl*’. We searched for all references up to August 9th, 2019. The results were limited to those articles referring to measurements of airborne mite-allergen concentration inside homes and written in the English language. References were selected on the basis of their abstracts. We also screened for descriptions of the measurements as well as details of the indoor environment. Data were extracted for the airborne concentrations, the measuring periods, and the particle size distribution. The procedure is illustrated in Fig. 1^20^. For this summary of statistics from the literature, we focussed on three characteristics of exposure: the mean concentration during undisturbed conditions, the mean concentration during disturbed conditions, and the peak concentration. The mean is defined as “the arithmetic average of the observations”^21^. Thus, the mean-value can differ depending on measuring during various types of human activity: for instance, when measuring both under disturbed and undisturbed conditions. Only articles were included reporting time-related statistics or statistics categorized to different indoor conditions.

### Measurements on particulate matter from beddings

In order to study the emissions from a bedding site, we measured the airborne dust levels in a bedroom of each of two Dutch family homes after shaking a duvet vigorously once or twice. Consent was obtained from the two families to conduct the pilot-study in their homes. One duvet was 16 years old (synthetic), the other 4 years old (feather). Both bedrooms were unheated and unused, with all ventilation devices off and the windows closed. Every six seconds, counts of particles in the size-range 0.25 to 32 µm were collected by an aerosol spectrometer (Grimm 1.109). Extractions from the datasets were confined to the period of mechanical activation of the duvet. Only data from the coarse fractions (particle diameters > 2 μm) were used. A recent study has shown that large particles (> 6 μm) tend to be deposited mainly in the upper airway, whereas particles in the size range 2–6 μm are deposited in the central and small airways^19^. In another study, Brown et al. observed that effectively all particles ≤ 1 μm penetrate (or pass) the extra-thoracic region as well as the tracheobronchial region^22^. These results indicate that particle sizes ≤ 1 μm are not of relevance in allergic asthma.

Total mass concentrations were obtained from the particle counts (assuming the particles to be made of material with a density of 1 g/cm^3^). We assessed the peak concentration and the sum of concentrations using of two approaches. The first method yielded an estimated mean for predicting the sum of concentrations, from which we derived a 1% upper prediction bound Y_0.99_ = $\overline{Y}$  + t_0.01;n-1_ * S * √ (1 + 1/n) for predicting the peak concentration (Y_0.99_ is the 1% upper prediction bound; $\overline{Y}$ is the estimated mean; t is the t-value; S is the standard deviation; n is the sample size). The second method used a nonlinear model Y_t_ = β_0_ + β_1_*exp (− β_2_ * t), where t is the time in seconds; Y_t_ is the concentration at time t; β_0_ is a parameter representing the background concentration; β_1_ is a parameter representing the concentration at t = 0 s; and β_2_ is a parameter representing the decay or settling rate of the dust concentration^4^. We assessed the quality of the fit by the coefficient of determination R^2^. A sensitivity-analysis yielded the fitting for the first ten minutes after activation of the duvet. All calculations were performed in R, version 3.4.1^23^. The package minpack.lm was used for estimating the nonlinear model.

## Results

### Description of mite aeroallergens concentration in the literature

We found 610 references related to the measurement of airborne house dust mite allergen concentration, of which 81 appeared to be duplicated (Fig. 1). Fifty-seven full articles were selected for screening descriptions of the measurements. Twenty-eight studies reported on measurements of airborne dust mite exposure in the home environment. All of these summarized the measurements by use of the mean. Ten of these studies presented time-related results on indoor exposure, for instance after changing the bedding^5,9,13,16,24–29^ (Table 1). Five studies used a volumetric air sampler^5,9,13,16,27^, and one used an ionic sampler^24^. The other four studies used an intranasal sampler^25,26^ or a personal sampler^28,29^. The mean concentrations during undisturbed conditions ranged from 0 to 1.7 ng allergen/m^3^, and the mean concentration during disturbed conditions ranged from 0.3 to 190 ng allergen /m^3^. These measurements were presented in various units (Der p1, allergen, protein).

**Table 1:** Summary of house dust mite aeroallergen measurements and statistical analyses in selected studies.

Study	Sampling device	Activity	n	Mean exposure/concentration during undisturbed conditions (duration)	Mean exposure/concentration during disturbed conditions (duration)	Peak exposure/concentration (duration)
Blay, 1991	Cassella Mark II cascade impactor	Vacuum cleaning	7	< 0.3 ng/m^3^ in group I and II mite allergen (20–120 min)	68 ng/m^3^ in group I and 25 ng/m^3^ in group II (20–120 min)	NA
Curtis, 2003	Ionic Breeze Quadra (an ion-charging device)	Normal domestic activities ("normal" not specified)	44	< 0.01 ng/m^3^ in group I mite allergen (120 min)	± 20 ng/m^3^ in group I mite allergen (45 min)	NA
Gore, 2002	Intranasal air samplers	Lying in bed	12	3 halo counts for Der p1 + Der p2 (30 min)	79 halo counts for Der p1 + Der p2 (30 min)	NA
Poulos, 1999	Intranasal air samplers	Domestic activities, including lying in bed	2	0 particles containing Der p1 (10 min)	9 to 10 particles containing Der p1 (10 min)	NA
Sakaguchi, 1989	Portable air-sampler (KI-636 Dylec)	Disturbed conditions in the bedroom, including bedmaking	10	0.03 Der p1 ng/m^3^ (109–124 h)	30.9 Der p1 ng/m^3^ (40 min)	NA
Sakaguchi	Portable air-sampler (KI-636 Dylec)	During sleep without the disturbance of the bedmaking	6	0.01 Der p1 ng/m^3^ (4.9 – 9.3 h)	0.22 Der p1 ng/m^3^ (4.9 – 9.3 h)	NA
Swanson, 1985	Air-Sentinel	Changing and shaking of the bedding	1	1.7 ng protein/m^3^ (24 h)	189.9 ng protein/m^3^ (20 min)	736.1 ng protein/m^3^ (5 min)
Tovey, 1981	Anderson filter holder connected to a vacuum pump	Domestic activities	13	< 0.3 ng Der p1 (120 min)	< 0.3 to 30 ng Der p1 (45 min)	NA
Tovey, 2013	A portable air-pump carried on the shoulder	Domestic activities and in-transit activities	12	0.05 ng/m^3^ (8 h)	1.12 ng/m^3^ (120 min)	NA
Tovey, 2016	A small impaction collector worn on the shoulder	Domestic activities and in- transit activities	10	0.02 ng/m^3^ (27 min)	0.09 ng/m^3^ (7.3 h)	NA

*NA* not applicable, *n* sample size.

Only one study^13^ presented a peak concentration (736 ng house dust mite allergen/m^3^ after 5 min) rather than a mean. This study is particularly interesting because their measurements began with the changing and vigorous shaking of the bedding while measuring, and ran for 24 h. Mite antigen concentration (protein) was measured in five different particle sizes (< 0.8 μm; 0.8–1.4 μm; 1.4–2.3 μm; 2.3–4.1 μm; > 4.1 μm) after sampling for 5 min, 20 min, and 24 h. The concentration measured was 100% at 5 min, 34.1% after 20 min, and 0.2% after 24 h. Also, other studies showed large differences between disturbed and undisturbed conditions. For instance, de Blay et al.^16^ reported a ratio > 200 between both conditions, indicating a rapid settling of particles.

### Particulate matter from beddings

**Figure 2:**
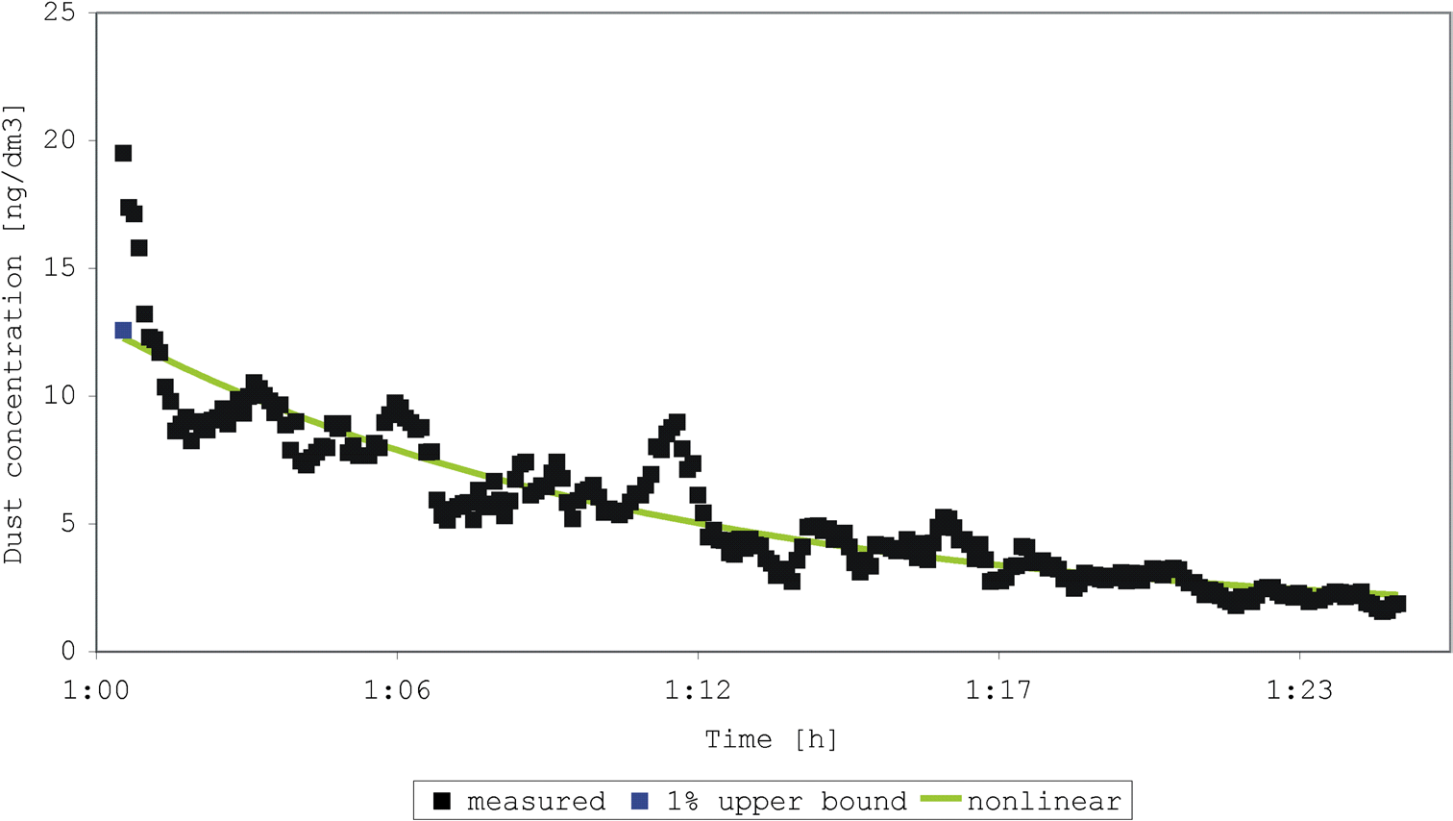
Measured and predicted dust concentration in experiment 1 after shaking a 4-year-old duvet one time.

**Figure 3:**
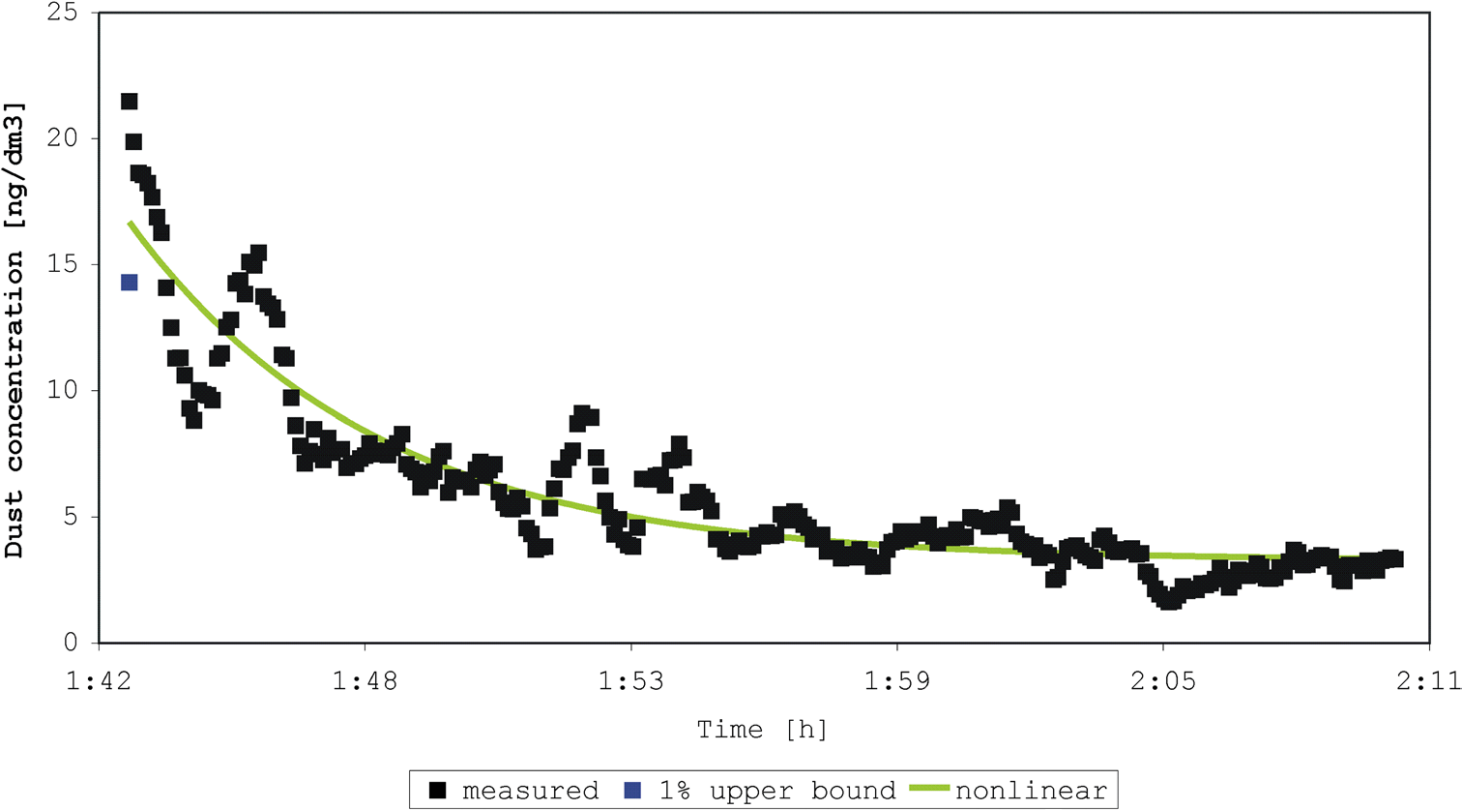
Measured and predicted dust concentration in experiment 2 after shaking a 4-year-old duvet two times.

**Figure 4:**
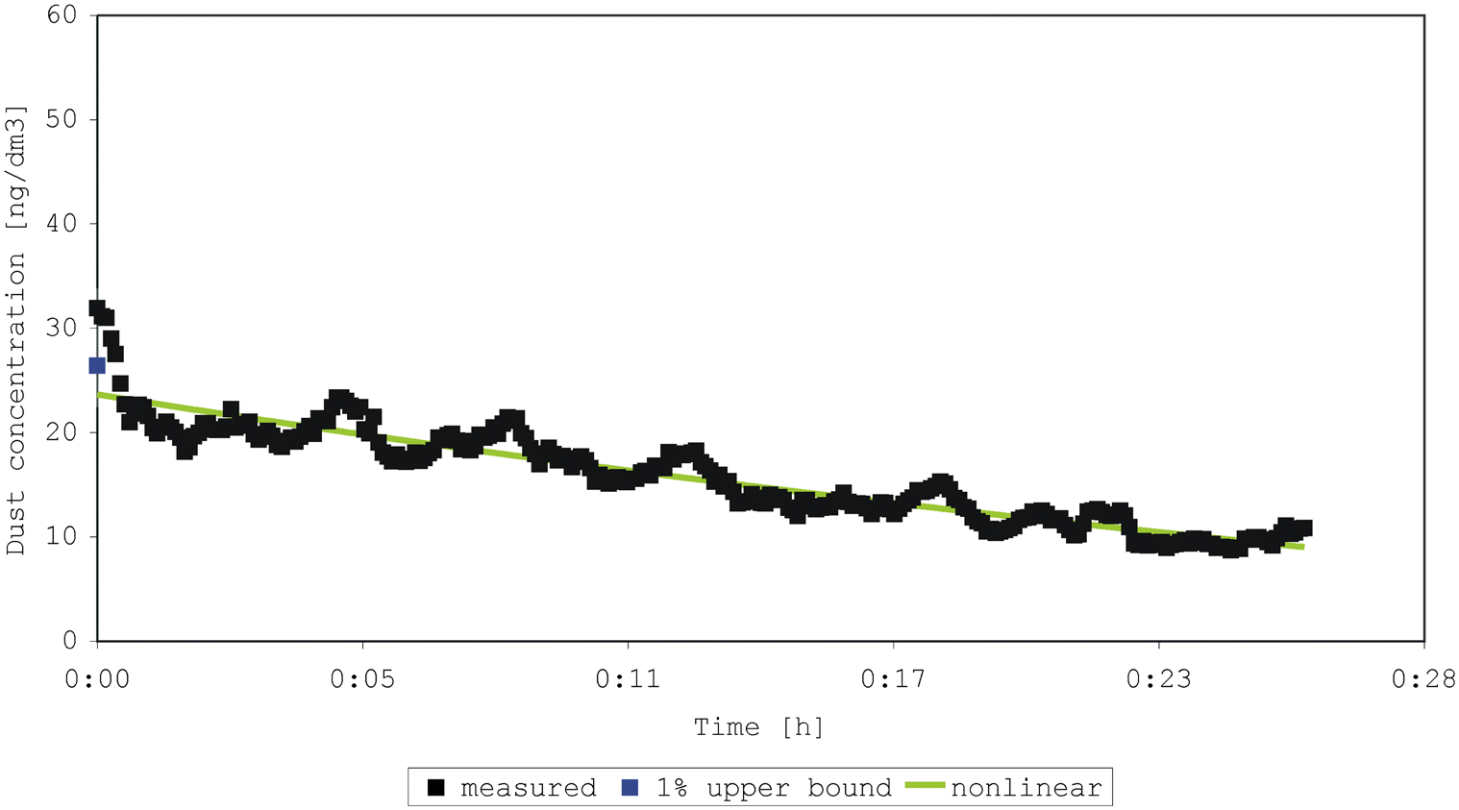
Measured and predicted dust concentration in experiment 3 after shaking a 16-year-old duvet one time.

**Figure 5:**
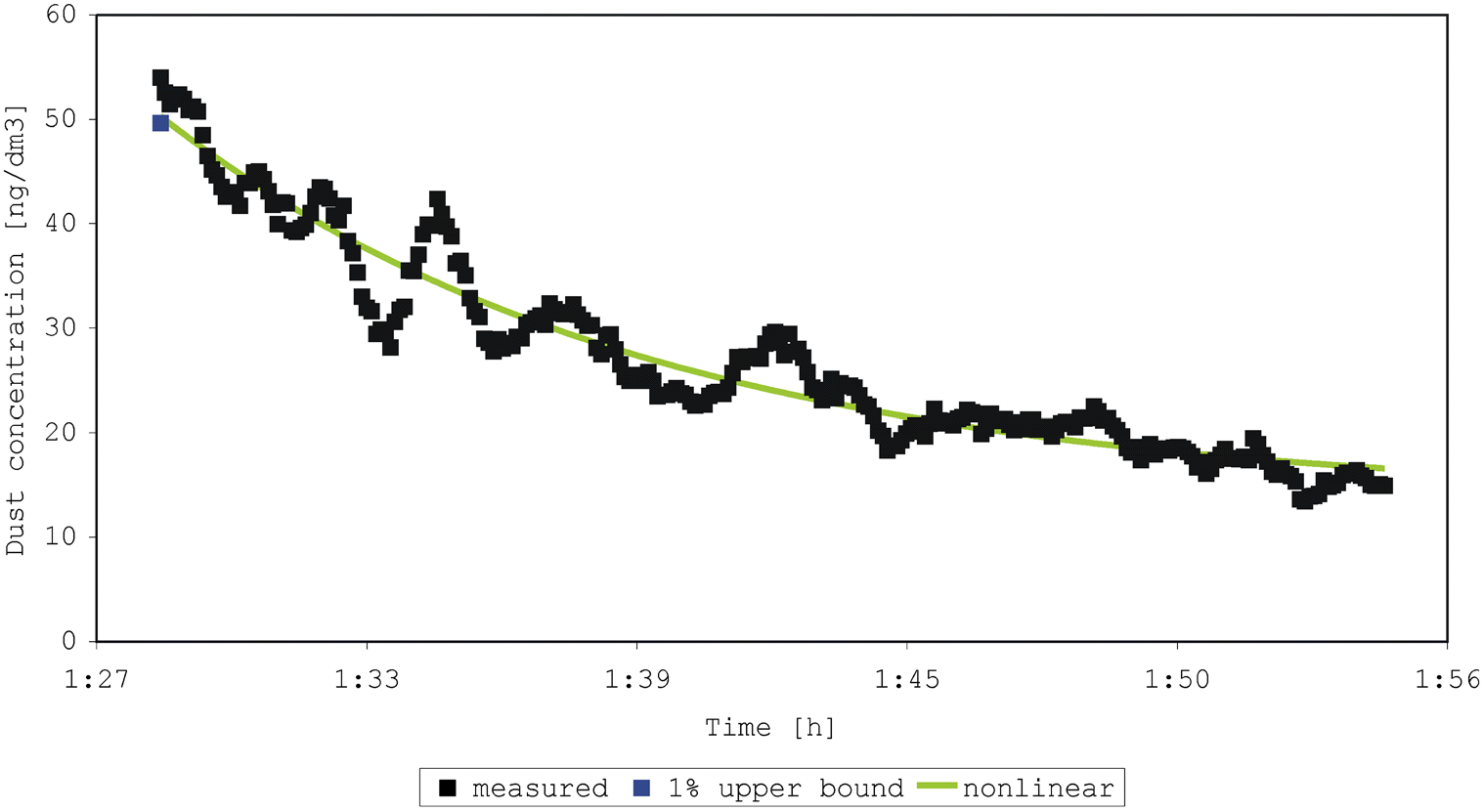
Measured and predicted dust concentration in experiment 4 after shaking a 16-year-old duvet two times.

The four experiments began with shaking a duvet one or two times (Figs. 2, 3, 4, 5). In each experiment, 245 to 275 measurements were made. The sum of concentrations in the experiments was 1337 to 7083 ng/dm^3^ dust, compared to an initial concentration of 19.5 to 54.0 ng/dm^3^. The older duvet (16 years) caused a higher initial exposure than the younger duvet (4 years). We achieved a perfect prediction (100%) of the sum of concentrations by both the estimated-mean method and the non-linear model for all four experiments. The percentage predicted initial concentration ranged from 64 to 92% (1% of the upper prediction bound), and 63 to 93% (nonlinear model) (Table 2). The coefficient of determination R^2^ for the four experiments was 0.09; 0.07; 0.05; 0.03 (use of the mean) and 0.85; 0.85; 0.86; 0.93 (nonlinear model).

**Table 2:** Predictions of the peak concentration measured from shaking a duvet (duration of measurement 6 s).

Experiment^a^	Shaking of the duvet (age of the duvet)	Initial or peak concentration (ng/dm^3^)	1% upper prediction bound (ng/dm^3^)	Prediction by non-linear model (ng/dm^3^)
1	One time (4 years)	19.5	12.6	12.3
2	Two times (4 years)	21.5	14.3	16.7
3	One time (16 years)	31.9	26.4	23.6
4	Two times (16 years)	54.0	49.6	50.3

^a^Experiment 1–4 shown in Figs. 2, 3, 4, and 5.

In a sensitivity analysis, we limited the data to the first ten minutes after activation of the duvet and found that the percentage predicted initial concentration ranged from 79 to 105% (1% of the upper prediction bound), and 88 to 106% (nonlinear model).

## Discussion

Measurement of personal mite aeroallergen exposure is complex. Earlier studies showed that human activities increased the mean aeroallergen levels^5,15,16^. The last development is the use of a small sampler, worn on the human body^29^. We investigated the way of describing the measurement of fluctuating indoor mite aeroallergen concentrations from beddings by comparing with summary-statistics of varying indoor particulate matter measurements.

A sample of fifty-seven articles on indoor measurements of airborne mite allergen exposure was taken from the literature. Only measurements in houses were considered. All articles summarized their results by the use of the mean (the arithmetic average of the observations), which is also sufficient to describe the sum of concentrations. Ten studies reported time-related indoor measurements, all including a mean during disturbed conditions and a mean during undisturbed conditions. A recent study on indoor aerosol dust particles suggests to measure fluctuations occurring during the disturbed and undisturbed conditions^6^. This is supported by the experiment by Swanson et al.^13^, who showed relative differences of a ratio of 110 between disturbed and undisturbed conditions. The peak concentrations measured by Swanson et al.^13^ was four times higher than the mean after 20 min measurement. These results should be interpreted with caution, as the assays used by Swanson et al.^13^ might vary considerably.

In our experiments also, peak values differed substantially from mean levels. Considering the differences between the measured emissions in our four experiments, shaking a duvet once or twice is not an easily reproducible disturbance. Again, however, the relative change was of importance in this case. Generally, the nonlinear model and a 1% upper bound predicted the peak level best when the variation in background exposure was low. Our data showed large fluctuations in the background levels, dominating the predicted decay after an emission, and reducing the quality of fit, especially for the nonlinear model. These large fluctuations can perhaps be explained by a heterogeneous distribution of the particles in the indoor air after the moment of emission. In general, the non-linear model fitted the data best (R^2^ ≥ 0.85 for data with large fluctuations). This fit improves when limiting the data to the first 10 min of measurement. The tendencies in the measurements by Swanson et al.^13^ are comparable with that of our experiments on particulate matter from beddings, highlighting the choice of using of tailored summary-statistics. However, the aim of our study was not necessarily to find the best predictive model, but rather the best way of describing the variation in the mite aeroallergen exposure.

The strength of our study is that, to our knowledge, this is the first study on how statistical principles should be applied to present results of airborne mite-allergen concentrations in combined disturbed and undisturbed conditions. Our pilot study showed tendencies consistent with the peak-decay found in the experiment by Swanson et al.^13^, indicating that the use of the mean alone is not sufficient to describe the fluctuating mite aeroallergen concentration from bedding. Multiple statistical models are available, like time series, a periodic function and regression^4,8^. Nevertheless, the wide ranges in reported results suggest that much more study of personal exposure is needed.

A major limitation of our study relates to the clinical implication. Clinically, it is clear that the increased allergen concentrations play a role in asthma symptoms^30^. However, it has yet to be confirmed whether asthma outcomes correlate with peak concentrations of house dust mite allergens. Laboratory experiments that have been performed on the relation between asthma outcomes and mite aeroallergen doses were mostly based on a homogeneous mite airborne dose^31–33^. Field studies in humans relating personal airborne mite-allergen levels to clinical symptoms of asthma are sparse. In 1996, Custovic et al.^34^ performed a study on the correlation between domestic mite allergen exposure and asthma severity in 53 patients during sleep. The overwhelming majority (94%) of mean airborne observations during the night were under the lower limit of detection for the allergen assay. Correlations were described between the allergen load and several asthma outcomes. While all the correlations were statistically significant, their magnitudes were all moderate (R^2^ = 0.38 to 0.49). These results show that more research is needed to understand the relationship between exposure and clinical outcomes. The use of tailored statistics combined with respiratory characteristics (e.g. FEV_1_/FVC), may allow the assessment of the actual aerosol exposure in the human airways, and provide evidence for the causal relation between house dust mite allergen exposure and allergic asthma in atopic patients.

In conclusion, measurements of indoor mite aeroallergen concentrations are commonly summarized by the mean. A comparison with fluctuating particulate matter measurements favours the use of peak exposure during disturbed conditions, calling for the use of other statistics than only the mean. We suggest that future studies describing mite aeroallergen measurements include information on the peak concentration as well as the mean. The measurements should be conducted with state of the art assay technology and more sophisticated mathematical models, such as regression or a time series analysis, should be used in the analysis.
